# Community Violence Exposure and Externalizing Problem Behavior Among Chinese High School Students: The Moderating Role of Parental Knowledge

**DOI:** 10.3389/fpsyg.2021.612237

**Published:** 2021-04-20

**Authors:** Yibo Zhang, Yuanyuan Chen, Wei Zhang

**Affiliations:** School of Psychology & Center for Studies of Psychological Application, South China Normal University, Guangzhou, China

**Keywords:** community violence exposure, deviant peer affiliation, externalizing problem behavior, parental knowledge, high school students

## Abstract

Adolescents' community violence exposure (CVE) has been demonstrated with a range of behavioral and psychological problems, but the processes that explain these correlations are not clear. In our 2017 study, the mediating role of deviant peer affiliation in the relationship between CVE and externalizing problem behaviors has been confirmed. However, the moderating effect of parental factors is still unclear. Therefore, a new group (high school group) was adopted in this study to further explore the moderating effect of parental knowledge based on also confirming the mediating effect of deviant peer affiliation. Stratified-cluster sampling was used to recruit 1,797 volunteers who completed questionnaires on CVE, deviant peer affiliation, parental knowledge, and externalizing problem behaviors. The results of the structural equation modeling were: on the basis of our previous research, we further analyzed the mediating role of deviant peer affiliation, and the mediated association was moderated by parental knowledge. Especially when the school climate is added as a covariate, the moderating effect of parental knowledge has changed, that is, the positive association between CVE and externalizing problem behaviors was much stronger for adolescents who reported lower levels of parental knowledge than for those who reported higher levels of parental knowledge. The results support the assumptions of social learning theory and have implications for interventions of community violence.

## Introduction

Community violence takes many forms, including shootings, fights between individuals or between gangs, and bombings. These forms of violence are intentional, are unpredictable, and occur in public (Elsaesser, 2018). Community members are exposed to violence through knowing the victims, witnessing violence, or being victimized. Osofsky (1995) defined community violence exposure(CVE) as “… frequent and continual exposure to the use of guns, knives, and random violence.” Children and adolescents are among the victims of community violence. According to the US National Survey of Children's Exposure to Violence II (NatSCEV II), the children and young people surveyed accounted for ~57.7%, and had undergone or witnessed at least one in five examples of violence (Finkelhor et al., 2013). In Chinese samples, Luo et al. (2006) found that 33.8% of middle school students suffered from violence imposed by other people.

Adolescent community violence exposure has been regarded as a major public health issue (Lambert et al., 2005). Carey (2012) made a detailed distinction between these two types of community violence exposure in their research, pointed out that witnessed CVE is considered to be the indirect exposure of an adolescent to a violent environment, such as seeing someone being attacked, robbed, or killed; suffered CVE corresponds to young people that have been directly attacked by violence, such as individuals being attacked or robbed. In recent years, more and more researchers have pointed out that heard violence is also a situation of indirect violence exposure, which means that the individual hears or knows that someone has been robbed or attacked (Richters and Saltzman, 1990; Davis et al., 2015). Studies have found that community violence exposure in adolescence is involved in a series of behavioral and psychological problems, such as aggression (Goodearl et al., 2013; Esposito et al., 2017; Tache et al., 2018), drug and alcohol abuse (Zinzow et al., 2009; Löfving-Gupta et al., 2018), and decline in academic performance (Taylor and Kliewer, 2006; Hardaway et al., 2014; Menard et al., 2015).

Problem behaviors are sometimes grouped as internalizing or externalizing problems (Jessor, 1991). Externalizing problem behaviors include problems such as rule-breaking and aggression. Although there is evidence for both, there is more evidence for externalizing problem behaviors; based on social learning theory (Bandura, 1973), we would expect a stronger direct effect between community violence and externalizing (through modeling and imitation) than between community violence and internalizing. Several studies have reported the negative influence of CVE on externalizing problems (Taylor and Kliewer, 2006; Conners-Burrow et al., 2013; Hardaway et al., 2014; Menard et al., 2015; Tien et al., 2019). Deane et al. (2018), for instance, found that there was a significant direct association between CVE (i.e., witnessing or victimization) and subsequent externalizing behaviors (i.e., aggression or delinquency) in their 2-year longitudinal study. Moreover, children exposed to long-term community violence exposure tended to show more aggressive behavior (Esposito et al., 2017).

Since high school students are used as samples in our study, we have to consider school factors (e.g., school climate) that may affect the reliability of the research results. Although a substantial body of previous studies have demonstrated that CVE has a significant impact on adolescent problem behavior, they have ignored the fact that school factors may bias the results (Fleckman et al., 2016; Zhang et al., 2017; Cooley et al., 2019). High school students spend most of their time in school every day, so when studying the impact of risk environment (e.g., CVE) on behavioral development, one cannot ignore the possible impact of school factors in it. Therefore, in this study, we included the school climate as a very important covariate in the analysis to reveal the impact of CVE on adolescents externalizing problem behaviors as objectively and scientifically as possible.

It is worth noting that in our 2017 study (Zhang et al., 2017), we confirmed the mediating effect of deviant peer affiliation on the relationship between CVE and externalizing problem behaviors by adopting a group of middle school students, and this mediating effect was moderated by the parent-child relationship. However, the moderating effect of the parent-child relationship was not interfered by external factors (e.g., school climate). Parental knowledge is related to adolescent behavior control and has a more direct impact on adolescent behavior development. Therefore, we hypothesize that parental knowledge is more susceptible to external interference (e.g., school climate), which changes its moderating effect in the mediation mechanism. Similarly, based on social learning theory (Bandura, 1973) and ecological theory (Bronfenbrenner, 1986), we tested whether the mediation mechanism of deviant peer affiliation in the relationship between CVE and externalizing problem behaviors was moderated by parental knowledge by choosing a different group (high school students). Moreover, after adding school climate, whether the moderating effect of parental knowledge has different results at different levels.

### Deviant Peer Affiliation as a Mediator

Peer groups can have positive or adverse impacts on the psychological and behavioral development of their members (Hartup, 1996). Deviant peer affiliation means the relation between deviant peers involved in interpersonal violence, crime, and substance abuse (Fergusson and Horwood, 1999; Zhu et al., 2016). Community violence exposure may increase the risk that adolescents will affiliate with deviant peers, leading to increased adolescent externalizing and even delinquent behavior (Hawkins and Weis, 1985). In other words, the community's socializing influence on the adolescent occurs in part through peer socialization processes. Previous studies have demonstrated the association between CVE and affiliation with deviant peers (Lambert et al., 2011; Low and Espelage, 2014; Liang et al., 2019). Moreover, according to Lambert et al. (2011), children with high levels of deviant peer affiliation are also highly likely to have had community violence exposure.

According to Bandura (1973) social learning theory, people learn new behaviors by observing and imitating the behavior of the people around them. When adolescents associate with deviant peers, they may over time learn aggressive and anti-social behaviors by observing and imitating other members of their peers. They may even come to consider these behaviors as legitimate (Bandura, 1973; Lin et al., 2020). There is substantial research showing an increasing risk of displaying externalizing problem behaviors when adolescents are affiliated with deviant peers (Chen et al., 2015, 2016; Hinnant et al., 2016; Tien et al., 2019). Chen et al. (2015) suggested in a review that affiliation with peers who engage in deviant behavior is always related to the development and maintenance of behavior problems. According to a longitudinal study of Hinnant et al. (2016), children's affiliation with deviant peers directly indicated that problem behavior is on the increase (e.g., externalizing symptoms, alcohol use, and marijuana use). Likewise, Tien et al. (2019) found that the number of affiliations with deviant peers predicted adolescents' risk of aggressive behavior.

Moreover, deviant peer affiliation appears to exert a mediating effect in the association between CVE and adverse outcomes in adolescence (e.g., antisocial behavior, low academic achievement). A review by Ingoldsby and Shaw (2002) showed that exposure to neighborhood violence significantly increased the number of deviant peers, which in turn significantly predicted the antisocial behavior of adolescents. Borofsky et al. (2013) demonstrated in a longitudinal study that deviant peer affiliation plays a mediating role in the relationship between CVE and children's study achievements. Our previous study showed that deviant peer affiliation had a significant mediating role between CVE and externalizing problem behaviors (Zhang et al., 2017). Moreover, recent studies have further confirmed our conclusion. For example, Liang et al. (2019) found that affiliating with risk-taking peer groups could significantly mediate the relationship between CVE and internet gaming disorder. Lin et al. (2020) found that deviant peer affiliation significantly mediated the association between CVE and aggressive behavior. Hence, in order to make our existing results more reliable (Zhang et al., 2017), we used a different group (high school students) in this study to further confirm the mediating effect of deviant peer affiliation on the relationship between CVE and externalizing problem behaviors, and to generalize our results.

### Parental Knowledge as a Moderator

Even though CVE serves as a general risk factor for deviant peer affiliation and externalizing behavior problems, many adolescents tend to produce more resilient outcomes than others. Consequently, when examining the relation among CVE, deviant peer affiliation, and externalizing behavior problems, protective factors should be taken into consideration. Parental knowledge, also known as parental behavioral control or parental monitoring, generally means a series of inter-correlated parenting behaviors related to being concerned of and tracking the activities, associates, friends, and whereabouts of their children (Dishion and McMahon, 1998). In fact, while parenting style and parent-child relationships have been extensively studied, parental knowledge about the whereabouts and activities of adolescents, that is, the influence of parental knowledge on adolescent behavioral development, has attracted more and more attention (Soenens et al., 2006; McAdams et al., 2014).

Parental knowledge function as a moderator has been elucidated in prior literature. Does parental knowledge moderate the path from CVE to externalizing problem behaviors? Based on the ecological systems theory and risk-buffering model (Cohen and Wills, 1985; Bronfenbrenner, 1986; Luthar et al., 2015), parental knowledge has received much attention as a positive impact on adolescents lives. Indeed, parental knowledge can decrease CVE (Udell et al., 2017; Liang et al., 2019), deviant peer affiliation (Marceau and Jackson, 2017), and externalizing problem behaviors (Jantzer et al., 2015; Chang and Qin, 2018). In a longitudinal study, the effect of CVE on adolescent delinquency was mitigated by parental monitoring, including deviant peer involvement (Low and Espelage, 2014). Furthermore, Schofield et al. (2015) found that parental knowledge could not only buffer the risks brought by deviant peers, but also further reduce the anti-social behavior of adolescents. Elam et al. (2017) found that parental monitoring had a moderating effect on the relationship between deviant peer affiliation and adolescents' substance use. Similarly, Lin et al. (2018) found that parental knowledge could moderate the association between risk-taking peer groups and internet gaming disorder. However, their research did not consider the influence of school factors on the moderating effect of parental monitoring, that is, they did not clarify the buffering effect of parental knowledge at different levels (high or low). Therefore, we think that the effect of parental knowledge as a moderator in amplifying adverse environmental risk (e.g., community violence exposure) on adolescent externalizing problem behavior *via* deviant peer affiliation remains to be further explored. Based on this literature, we proposed the hypotheses as follows:

**Hypothesis 1**. Parental knowledge has a moderating effect on the process by which CVE is associated with adolescent externalizing problem behaviors *via* affiliation with deviant peers. Specifically, high parental knowledge might reduce the significant positive effects of community violence exposure and deviant peer affiliation on externalizing problem behavior. Meanwhile, high parental knowledge might buffer the effects of deviant peer affiliation on externalizing problem behavior.

**Hypothesis 2**. If school climate is included as a covariate in the analysis, different levels (low level and high level) of parental knowledge might show different moderating effects at different stages of the mediating effect.

### The Present Study

**Figure 1:**
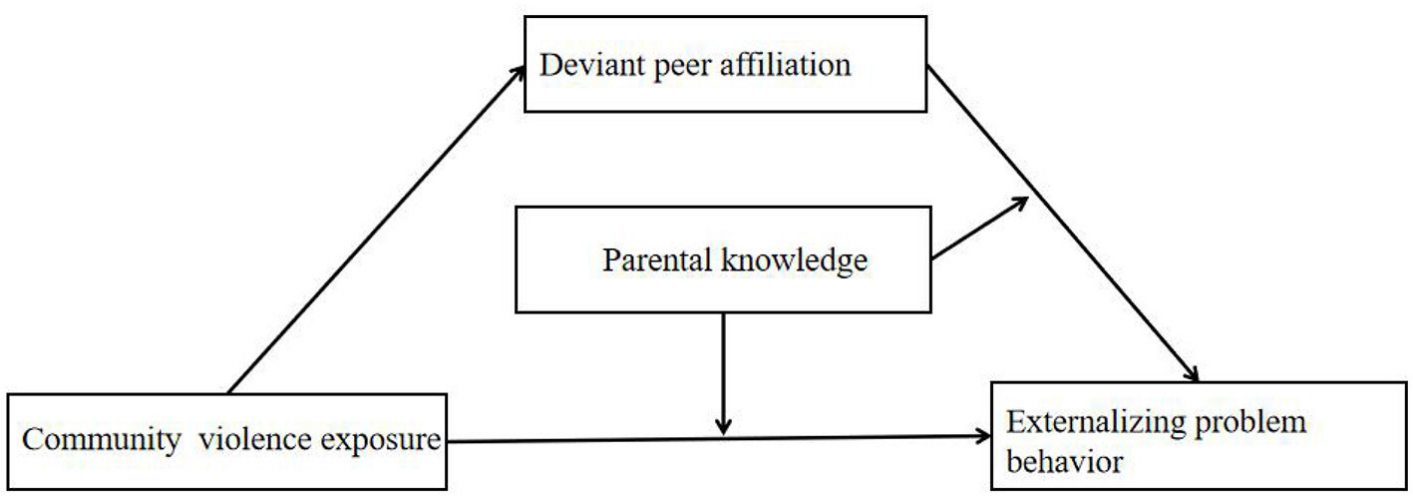
Conceptual framework of the proposed moderated mediation model.

Our previous study has shown that CVE in adolescent externalizing problem behaviors and deviant peer affiliation has a significant mediating role (Zhang et al., 2017). However, there are still some questions that have not been fully explored. For example, (1) whether deviant peer affiliation still has a significant mediating effect in another group (e.g., high school students), and whether other parental factors (e.g., parental knowledge) also play a moderating role; (2) although the influence of school factors (e.g., school climate) on the results was noted in our previous studies (Zhang et al., 2017), we did not further compare whether the moderating effect would change after the addition of school climate and whether the school climate would affect the moderating effect at different stages of the mediation. Therefore, this study expands the existing literature on Chinese adolescents' community violence exposure and externalizing problem behaviors. We hypothesized that: (a) parental knowledge has a moderating effect on the process by which CVE is associated with adolescent externalizing problem behaviors *via* deviant peer affiliation; (b) after adding school climate as a covariate, different levels of parental knowledge will show different moderating effects. The proposed research model is shown in Figure 1.

## Materials and Methods

### Participants

A total of 1,797 high school students (45.5% male, mean age = 16.07, SD = 0.99) were selected from four secondary schools in Guangdong Province, China by stratified and random cluster sampling. About half of the adolescents' parents had an education level of less than high school (25.0% of fathers; 14.3% of mothers). Among them, 58.7% of families were from rural areas, 1.8% were from small and medium-sized cities, and 12.6% were from metropolitan areas. The majority of adolescents (69.4%) came from families with average monthly earnings of RMB 0 to 2,000.

### Measures

#### Community Violence Exposure

The Chinese version of the CVE questionnaire (Zhang et al., 2017) was used to measure CVE, which includes six items. Each item requires respondents to show how often they have heard any of the six forms of violence (i.e., people being robbed) in the neighborhood on a 5-point scale of 1 (never) to 5 (always) in the past six months. The composite score was gained through calculating the average of the six items; the higher the score, the higher the community violence exposure. The sample was subjected to confirmatory factor analysis to obtain the following fitting index: χ^2^ = 53.008, df = 6, *p* < 0.001, TLI = 0.98, CFI = 0.97, RMSEA = 0.06, SRMR = 0.02. The results show that the questionnaire had acceptable construct validity. The Cronbach's alpha for this scale was 0.80.

#### Deviant Peer Affiliation

Adolescents used the Deviant Peers Questionnaire (Chinese version) to report their deviant peer affiliation (Zhu et al., 2015). A total of 12 items evaluated how many participants' peers showed deviant behaviors in the last 6 months. For instance, “In the past 6 months, how many of your friends were involved in a fight?” Each item was scored from 1 (never) to 3 (six or more times). By calculating the average of the 12 items, it was concluded that the higher the score, the higher the deviant peer affiliation. The sample was subjected to confirmatory factor analysis to obtain the following fitting index: χ^2^ = 935.789, df = 89, *p* < 0.001, TLI = 0.92, CFI = 0.90, RMSEA = 0.07, SRMR = 0.05. The results show that the questionnaire had acceptable construct validity. The Cronbach's alpha was 0.87.

#### Parental Knowledge

A Parental Knowledge Questionnaire with five items, which was utilized in the study of Jiang et al. (2016) and showed good validity and reliability in the previous research, was adopted. The respondents were required to report on how much their parents knew about how they spent their leisure time in the last 6 months. For instance, “Do your parents know what activities you do in your free time?”; “Do your parents know where you are going when you go out with friends at night?” Participants scored each item on a 3-point scale, from 1 (know a little) to 3 (know well). The average of all items was calculated, the higher the score, the higher the level of parental knowledge. The sample was subjected to confirmatory factor analysis to obtain the following fitting index: χ^2^ = 34.461, df = 4, *p* < 0.001, CFI = 0.986, TLI = 0.964, RMSEA = 0.065, SRMR = 0.02. The results show that the questionnaire had good construct validity. The Cronbach's alpha coefficient of this questionnaire was 0.77.

#### Externalizing Problem Behavior

Based on the previously published questionnaire (Zhu et al., 2016), six items were selected to measure the externalizing behaviors of adolescents. Participants were required to show the frequency they exhibited problem behaviors such as cheating, alcohol use, smoking, fighting, and excessive Internet use during the last half of the year. All the items were scored on a 5-point scale, from 1 (never) to 5 (six times or more). Through calculating the average of all items, it was concluded that the higher the score the higher the level of problem behavior. The sample was subjected to confirmatory factor analysis to obtain the following fitting index: χ^2^ = 929.302, df = 87, *p* < 0.001, TLI = 0.91, CFI = 0.90, RMSEA = 0.07, SRMR = 0.05. The results show that the questionnaire had good construct validity. The Cronbach's alpha coefficient of this questionnaire was 0.79.

#### School Climate

The adolescent perception of school climate questionnaire compiled by Jia et al. (2009) revised by Zheng et al. (2015) has a total of 25 items, including teacher support (such as my teachers care about me), peer support (such as mutual help among students), and opportunities for autonomy (such as students having a say in school matters), and was used in this study. All the items were scored on a 5-point scale, from 1 (never) to 5 (always). In view of the significant high correlation between teacher support, peer support, and opportunities for autonomy, the average score of all items was calculated. The higher the score, the more positive the young people's perception of the school climate. The sample was subjected to confirmatory factor analysis to obtain the following fitting index: χ^2^ = 1755.126, df = 253, *p* < 0.001, TLI = 0.90, CFI = 0.90, RMSEA = 0.06, SRMR = 0.06. The results show that the questionnaire had acceptable construct validity. The Cronbach's alpha coefficient of this questionnaire was 0.87.

#### Covariates

In view of the results of previous research showing that age, gender, and family socioeconomic status (SES) were related to the main variables in this study (Zhu et al., 2016), these demographic variables were then used as control variables in our statistical analysis. Based on Veenstra and Dijkstra (2011) study, the average of the respondents' standardized scores on four items (such as parent educational attainment, family monthly income per capita, and geographic area) can be used to measure SES. Considering the influence of school factors, we chose school climate as a covariate. Adolescent gender was represented by a dichotomous variable (1 = male; 0 = female). Participants were required to describe their geographical area on a 5-point scale, and the range was from underdeveloped to more developed (1 = rural area, 5 = metropolis). Educational attainment was measured using an 8-point Likert scale (1 = unschooled, 8 = doctor). Respondents' income was measured by the family's per capita income each month, which was a 10-category variable (1 = ≤ RMB 1,000, 10 = ≥RMB 9,001).

### Procedure

Data collection was carried out in the interviewee's classroom by a well-trained data collector (post-graduate in psychology). On the day of the questionnaire collection, our graduate research assistants personally went to the school to collect the questionnaire on site. The test lasted about 45 min. After the students completed the questionnaire, we took back the questionnaire and checked the basic information on the spot. Before collecting data, we first obtained the informed consent of the parents and the consent of the teenagers. Participants were told that their participation was voluntary, their privacy would be protected, and they could withdraw at any time during the data collection period. The research ethics committee of the corresponding author's institution approved all materials and procedures.

### Statistical Analyses

In the community violence exposure questionnaire, if the average value in the total items was ≥2 the adolescents were considered to have experienced CVE. According to this standard, the prevalence of ECV was 85.36%. Descriptive statistics were performed using SPSS 20.0. The structural equation modeling (SEM) methods with M-plus 7.0 (Muthén and Muthén, 1998-2012) were used to test the mediation and moderation effects (Preacher et al., 2007; Erceg-Hurn and Mirosevich, 2008). The formal mediation test was also performed using a bootstrapping procedure, which computed an estimation of the indirect effect with a 95% confidence interval (CI). Multiple fit indices, such as the ratio of chi-square to degrees of freedom (χ^2^/df), comparative fit index (CFI), and root mean square error of approximation (RMSEA), were used to assess the model fit. Some SEM literature showed that if χ^2^/df ≤ 3, CFI ≥ 0.95, and RMSEA ≤ 0.06, the model fit is good (Kline 2011; Hoyle 2012). The squared coefficients before standardization indicated that the common variance was 21.63% when the common latent factor (constructed by the path leading to each observed variable in the measurement model) was added, which was <40% threshold (Eichhorn, 2014). Therefore, our conclusion is that in the current study, common method deviations were unlikely to cause much concern.

### Data Availability Statement

The raw data supporting the conclusions of this article will be made available by the corresponding authors, without undue reservation, to any qualified researcher.

## Results

### Descriptive Statistics

The means, standard deviations, skewness, kurtosis, and the Pearson product-moment correlations are displayed in Table 1. As can be seen from Table 1, there was a significant positive correlation between CVE and externalizing problem behaviors, indicating that CVE might be a risk factor for adolescent problem behaviors. Moreover, there was a positive correlation between externalizing problems and deviant peer affiliation. However, parental knowledge was significantly negatively correlated with adolescent externalizing problem behavior and deviant peer affiliation.

**Table 1:** The Pearson product-moment correlations for all variables.

**Variable**	**1**	**2**	**3**	**4**	**5**	**6**	**7**	**8**	**9**	**10**
Age	1.00									
Gender	0.02	1.00								
School climate	−0.02	−0.08^**^	1.00							
Family income status	−0.14^***^	0.04	0.03	1.00						
Father's education level	−0.22^***^	0.02	0.11^***^	0.27^***^	1.00					
Mother's education level	−0.27^***^	0.02	0.13^***^	0.35^***^	0.57^***^	1.00				
Community violence exposure	0.02	0.08^**^	−0.21^***^	−0.00	−0.05^*^	−0.05^*^	1.00			
Deviant peer affiliation	0.00	0.18^***^	−0.18^***^	−0.01	−0.04	−0.04	0.22^***^	1.00		
Parental knowledge	−0.03	−0.18^***^	0.27^***^	−0.02	0.11^***^	0.12^***^	−0.11^***^	−0.19^***^	1.00	
Externalizing problem behavior	0.00	0.16^***^	−0.16^***^	0.08^*^	0.00	−0.03	0.21^***^	0.35^***^	−0.21^***^	1.00
*M*	16.07	—	3.43	2.07	2.99	2.23	1.72	1.39	2.24	1.07
SD	0.99	—	0.47	1.58	1.03	1.22	0.60	0.63	0.55	0.23
SK	0.52	—	−0.15	2.11	1.01	1.26	1.36	2.27	−0.40	2.88
KU	1.71	—	0.53	4.76	2.15	1.87	3.30	5.99	−0.67	8.05

*Gender and age were dummy coded: 1 = male, 0 = female; SK, skewness; KU, kurtosis*.

* *p < 0.05*,

** *p < 0.01*,

*** *p < 0.001*.

### Test for the Mediating Effect of Deviant Peer Affiliation

**Figure 2:**
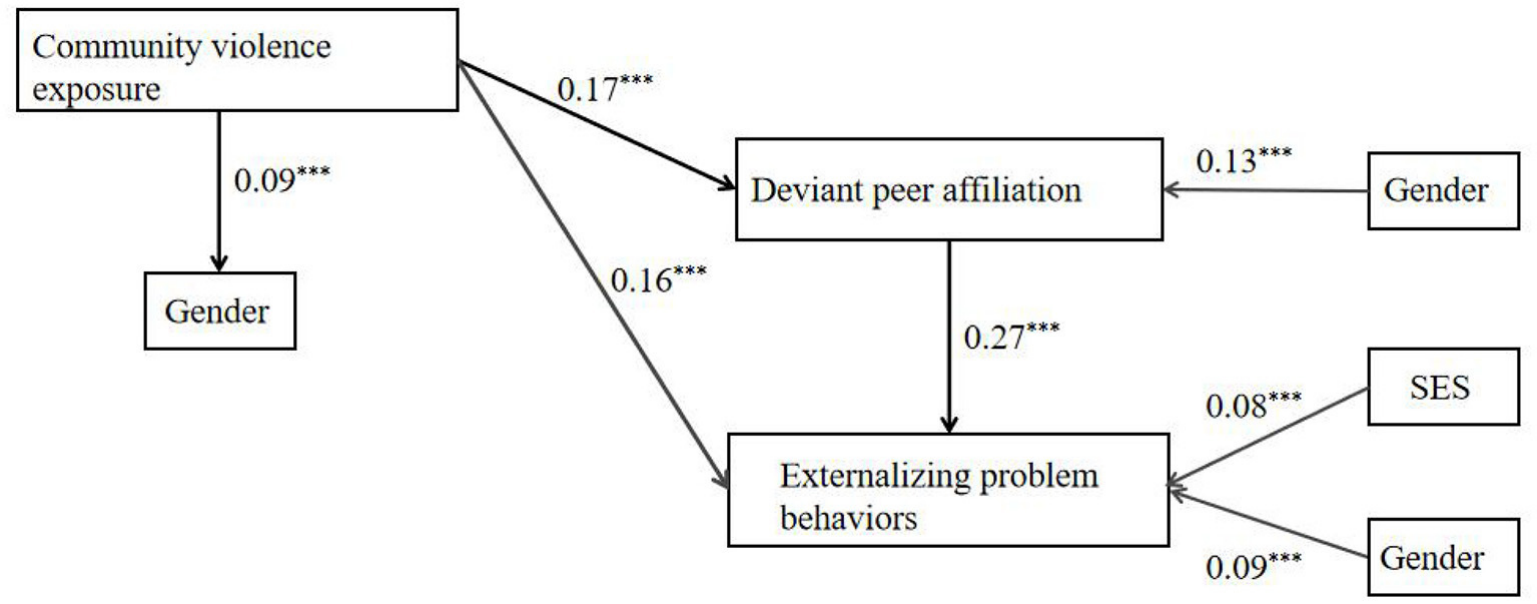
Model of the mediating role of deviant peer affiliation between community violence exposure and externalizing problem behaviors. ^***^p < 0.001.

Although we have already analyzed the mediating effect of peers, a high school group (different from the middle school group in the 2017 study) was used in this study, so it was necessary to test the mediating effect again to verify the stability of our previous results. In the mediation model in Figure 2, deviant peer affiliation mediated the association between CVE and externalizing problem behavior (χ^2^/df = 0.00, TLI = 1.00, CFI = 1.00, RMSEA = 0.00). Moreover, community violence exposure was positively associated with adolescents' externalizing problem behavior (*b* = 0.16, *p* < 0.001), and there was a positive association between deviant peer affiliation and community violence exposure (*b* = 0.17, *p* < 0.001). The path from deviant peer affiliation to externalizing problem behavior was also significant (*b* = 0.27, *p* < 0.001), with high levels of deviant peer affiliation predicting more problem behaviors. Bootstrapping analysis showed that in the association between CVE and externalizing problems, deviant peer affiliation had a significant positive mediating effect (indirect effect *t* = 0.046, SE = 0.014, *p* < 0.001, 95% CI [0.020, 0.071]).

### Test for Moderated Mediation

#### Moderating Effects of Parental Knowledge on the Direct Association Between CVE and Externalizing Problem Behavior

**Figure 3:**
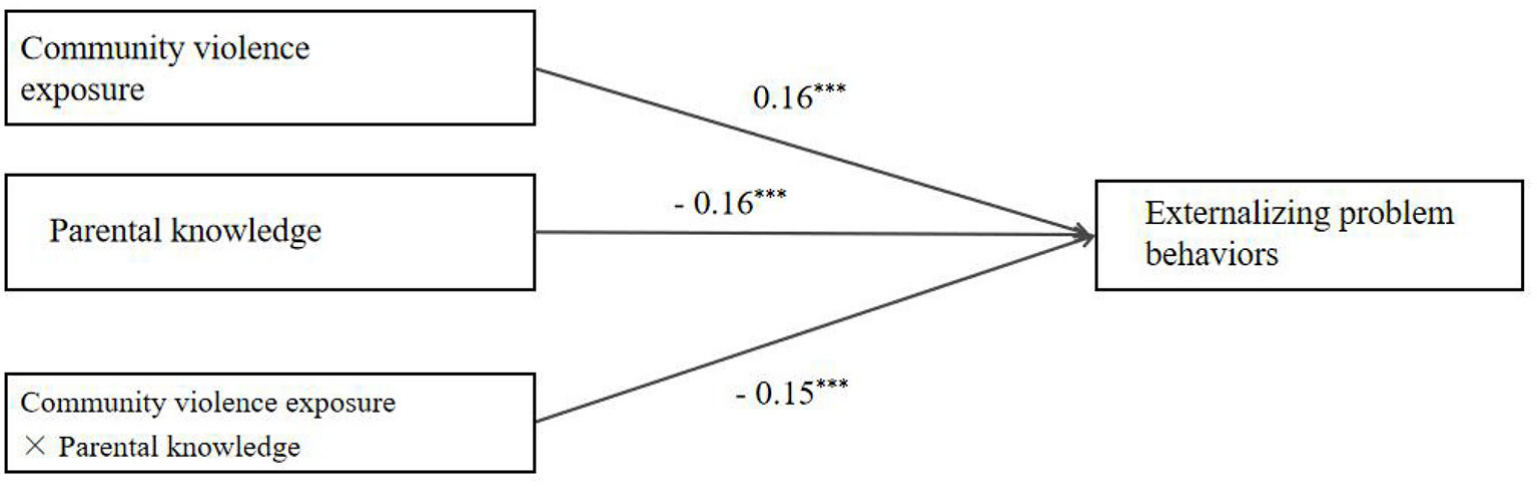
Model of the moderating role of parental knowledge on the direct association between community violence exposure and externalizing problem behaviors. ^***^p < 0.001.

It can be seen from the moderated model (see Figure 3) that there was a good fit between the model and the data (χ^2^/df = 2.25, CFI = 0.97, TLI = 0.92, RMSEA = 0.03, SRMR = 0.01). Both CVE (*b* = 0.16, 95% CI [0.089, 0.025]) and parental knowledge (*b* = −0.16, 95% CI [−0.222, −0.113]) significantly predicted externalizing problem behaviors. Furthermore, the interaction effect between CVE and parental knowledge on externalizing problem behavior was also significant (*b* = −0.15, 95% CI [−0.332, −0.025]).

**Figure 4:**
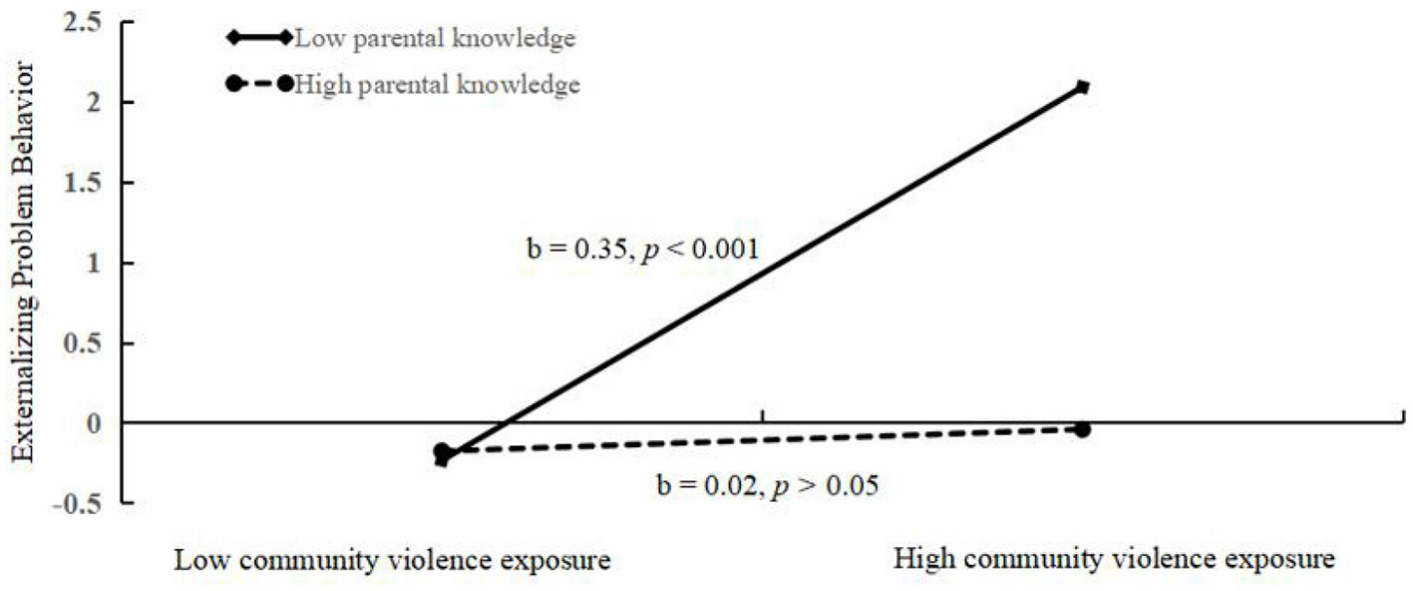
Association between community violence exposure and externalizing problem behaviors at higher and lower levels of adolescent parental knowledge (Before add covariate: school climate).

**Figure 5:**
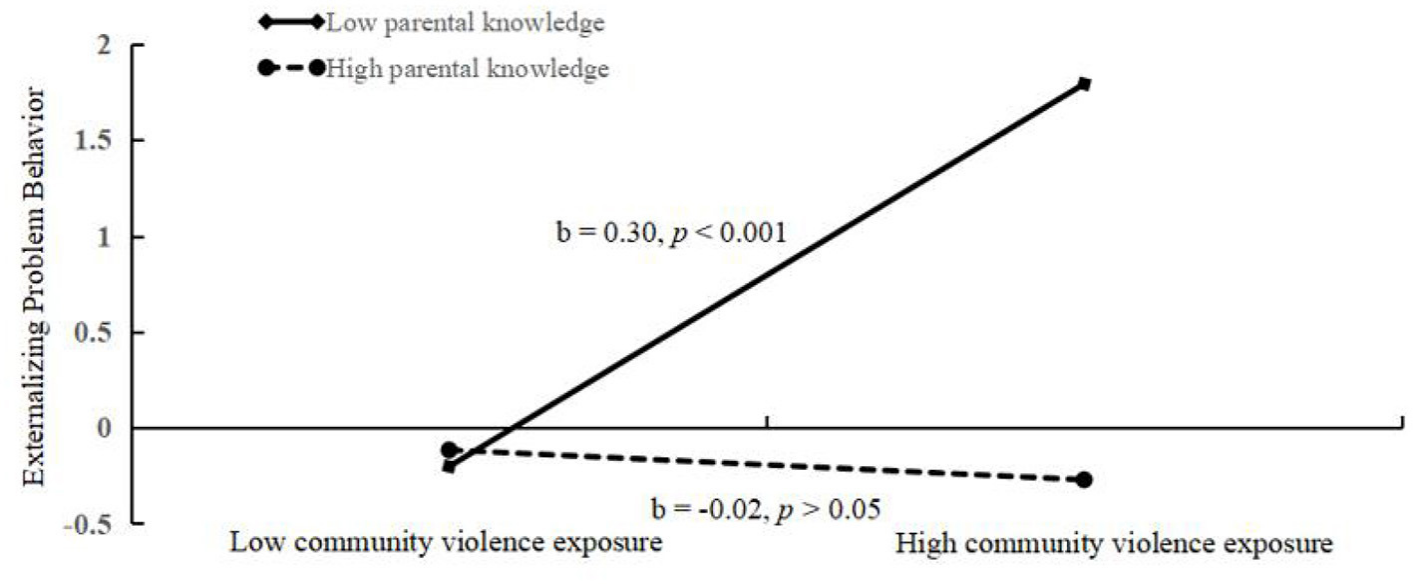
Association between community violence exposure and externalizing problem behaviors at higher and lower levels of adolescent parental knowledge (After add covariate: school climate).

We conducted a simple slopes test before (Figure 4) and after (Figure 5) adding covariates. Only when adolescents reported lower levels of parental knowledge, the positive relationship between CVE and externalizing problem behavior was significant (−1 SD: Figure 4: *b* = 0.35, *p* < 0.001; Figure 5: *b* = 0.30, *p* < 0.001). On the contrary, a positive relationship between CVE and externalizing problem behavior was not significant for adolescents, who reported high levels of parental knowledge (+1 SD: Figure 4: *b* = 0.02, *p* > 0.05; Figure 5: *b* = −0.02, *p* > 0.05).

#### Test for the Moderating Effect of Parental Knowledge on the Indirect Association Between Community Violence Exposure and Externalizing Problem Behavior

**Figure 6:**
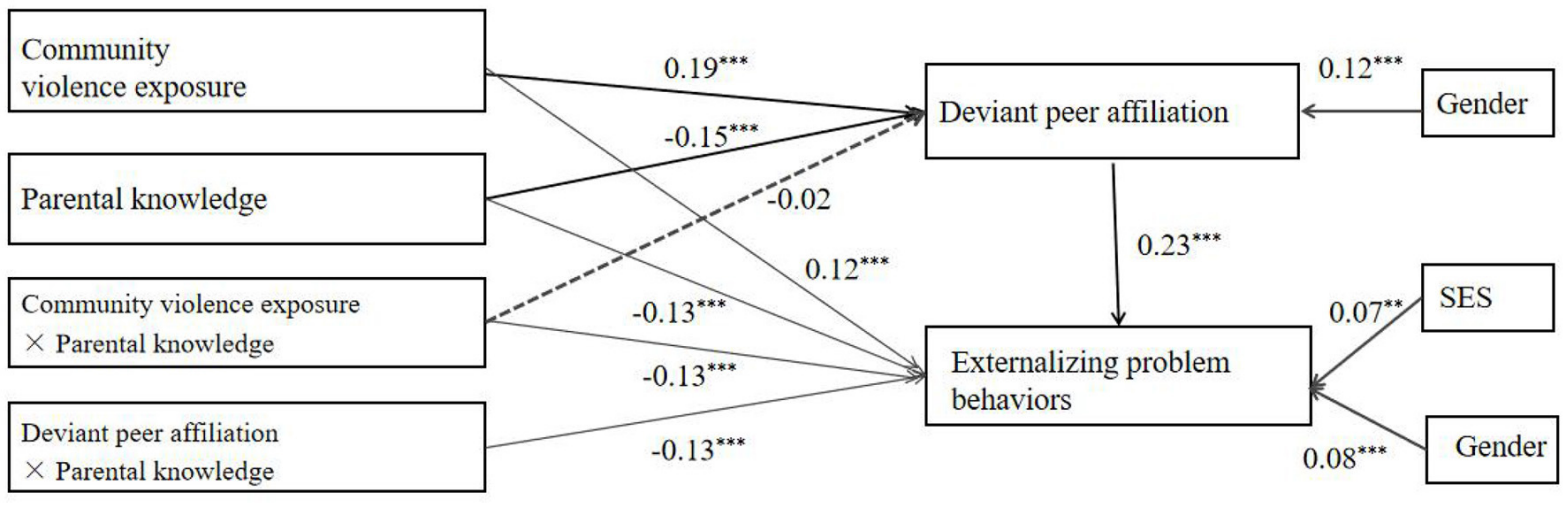
Test of the moderating effect of parental knowledge on the indirect association between community violence exposure and externalizing problem behaviors. ^***^p < 0.001, ^**^p < 0.01.

**Figure 7:**
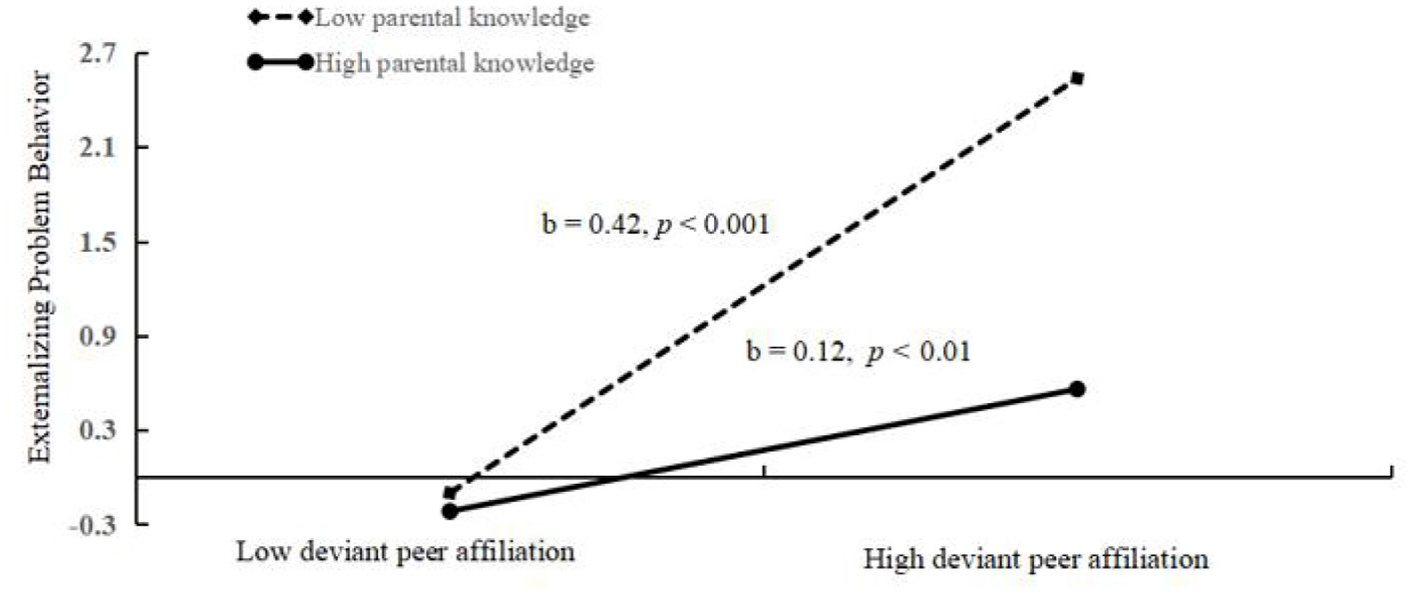
Association between deviant peer affiliation and externalizing problem behaviors at higher and lower levels of adolescent parental knowledge (Before add covariate: school climate).

**Figure 8:**
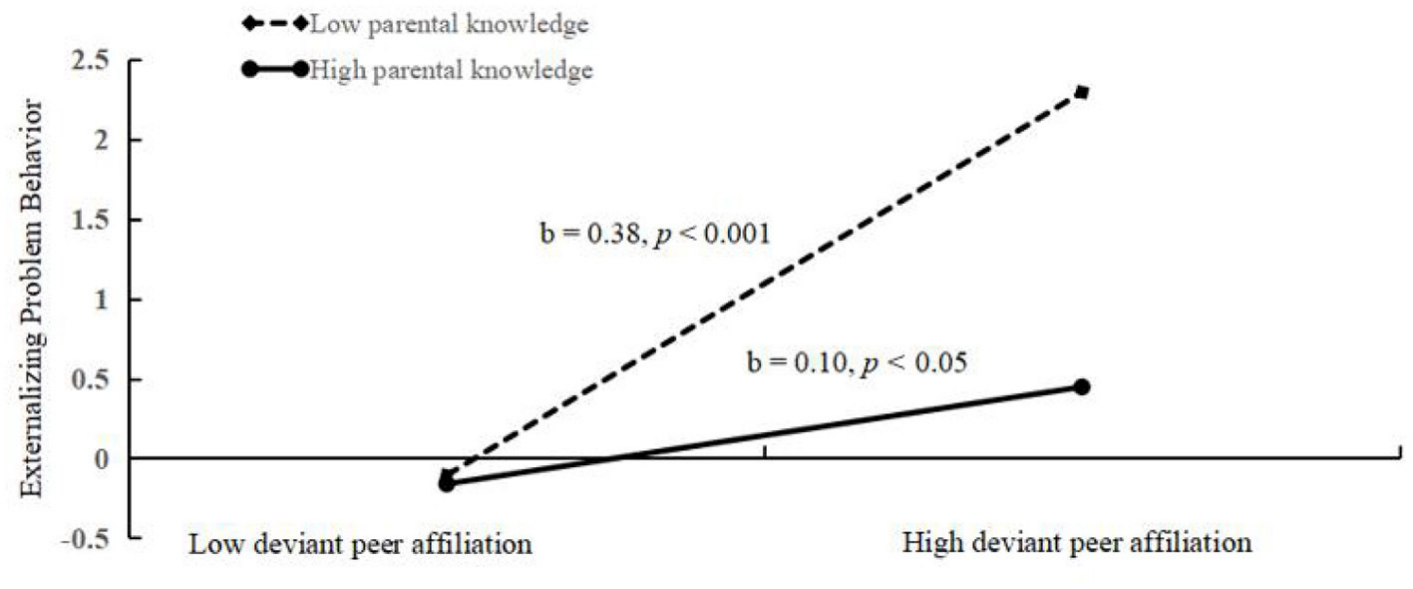
Association between deviant peer affiliation and externalizing problem behaviors at higher and lower levels of adolescent parental knowledge (After add covariate: school climate).

It can be seen from the moderated mediation model (see Figure 6) that there was a good fit between the model and the data (χ^2^/df = 2.00, CFI = 0.98, TLI = 0.96, RMSEA = 0.024, SRMR = 0.015). The estimates and test statistics result in different paths including the interaction effect of deviant peer affiliation and parental knowledge. Both deviant peer affiliation (*b* = 0.23, *p* < 0.001) and parental knowledge (*b* = −0.13, *p* < 0.001) significantly predicted externalizing problem behaviors. Furthermore, parental knowledge functioned as a moderator in the relationship between deviant peer affiliation and externalizing problem behavior (*b* = −0.13, *p* < 0.001). Moreover, both CVE (*b* = 0.19, *p* < 0.001) and parental knowledge (*b* = −0.15, *p* < 0.001) significantly predicted deviant peer affiliation, but the relationship between CVE and deviant peer affiliation was not moderated by parental knowledge (*b* = –0.02, *p* > 0.05). Moreover, as depicted in Figure 7, simple slope plots and calculations were conducted at −1 SD and +1 SD of the average of deviant peer affiliation. The result revealed that the association between deviant peer affiliation and externalizing problem behavior was significantly stronger in the context of lower parental knowledge (*b* = 0.42, *p* < 0.001) than that in the context of higher parental knowledge (*b* = 0.12, *p* = 0.008). On the contrary, after adding school climate as a covariate, the result (Figure 8) revealed that the association between deviant peer affiliation and externalizing problem behavior was significant in the context of lower parental knowledge (*b* = 0.38, *p* < 0.001), however, in adolescents with high level parental knowledge, the association was still significant but was weaker than before (*b* = 0.10, *p* = 0.030). Finally, the bias-corrected percentile bootstrap method was used to examine the conditional indirect effects of CVE on externalizing problem behavior as a function of parental knowledge. The results revealed that conditional indirect effects were found to be significant for adolescents with lower parental knowledge (*b* = 0.0645, SE = 0.0177, 95% CI [0.0326, 0.1009]) and for adolescents with higher parental knowledge (*b* = 0.0241, SE = 0.0141, 95% CI [0.0002, 0.0554]). Thus, parental knowledge was a protective factor between CVE and externalizing problem behavior.

Finally, in order to further clarify the influence of gender on the moderated mediation model proposed in this study, we used gender as a moderating variable for supplementary analysis. Based on the result of dual interactions (e.g., community violence exposure × gender → externalizing problem behaviors/ deviant peer affiliation, gender × deviant peer affiliation → externalizing problem behaviors) and triple interactions (e.g., community violence exposure × parental knowledge × gender → deviant peer affiliation/externalizing problem behaviors, deviant peer affiliation × parental knowledge × gender → externalizing problem behavior), all the moderating effects of gender were not significant, so in the discussion, no in-depth analysis of gender roles was conducted.

## Discussion

Although our 2017 study confirmed the mediating role of peer factors (e.g., deviant peer affiliation) in the relationship between community violence exposure and externalizing problem behaviors, which was consistent with previous studies (Goodearl et al., 2013; Hinnant et al., 2019), and the negative effect of CVE on externalizing problem behaviors in adolescents has been documented (Ziv, 2012; Lepore and Kliewer, 2013; Fagan et al., 2014; Stoddard et al., 2015; McGee et al., 2019), the moderating role of parental factors is still unclear. Although in our previous studies, we noted that school climate was a very important covariable for the moderating effect. However, we did not further compare its specific effects on the mediating mechanism. Based on these considerations, in this study, we used a different group (e.g., high school students) to further verify the mediating mechanism and further analyze the influence of school climate on the moderating effect.

The results of our current study showed that parental knowledge could moderate this indirect association in the second stage of the mediation process and the direct association between CVE and externalizing problem behavior. This study expands previous research by analyzing sample data of Chinese adolescents. The results highlight the complexity of understanding the association between CVE and externalizing problem behaviors in adolescents. Therefore, it is of great importance to explore the factors that exert influence on adolescents' exposure to violence in their communities and to develop targeted prevention and intervention methods.

### Moderating Effect of Parental Knowledge

The findings partially supported Hypothesis 1, that is, CVE interacts with parental knowledge to amplify the mediation processes. Firstly, our findings suggest that parental knowledge could significantly moderating the direct relationship between CVE and externalizing problem behavior. Specifically, this moderating effect was only significant for adolescents who reported a low level of parental knowledge. However, a high level of parental knowledge had no significant moderate effect on this direct association. Compared with previous studies (Liang et al., 2019), our findings further confirmed that adolescents with low-level parental knowledge were more likely to exhibit externalizing problem behaviors in a long-term environment of CVE.

Secondly, parent knowledge moderated the indirect relationship between CVE and externalizing problems *via* deviant peer affiliations, but this effect was only significant in the second stage of the mediation process (i.e., deviant peer affiliation → externalizing problem behavior). According to ecological systems theory (Bronfenbrenner, 1986), adolescents with a high level of positive family characteristics, such as parental knowledge, are unlikely to affiliate with deviant peers in high-risk environments (e.g., CVE). This means that adolescents with low-level parental knowledge are more likely to be friends with more deviant peers in a risky environment (e.g., CVE). On the contrary, for families with high-level parental knowledge, young people grow up in a more harmonious and warm family, and they have a more secure community environment and high-quality peers, thus showing a lower level of problem behavior. However, when parents are less aware of the activities and friendships of their children, serious harm can be caused by deviant peer groups, and a higher number of problem behaviors would eventually occur. For instance, Narayan et al. (2015) found that under the condition of violence, parent warmth has a protective effect on children. When adolescents are exposed to violence at a high level but have a high level of parent warmth, there is a reduced likelihood of bullying peers or making friends with deviant peers. Similarly, Jiang et al. (2016), in a longitudinal study, found that parental knowledge could moderate the relationship between deviant peer affiliation and adolescents' alcohol use. However, our results failed to find a moderating role of parental knowledge in the first stage of the mediation process (community violence exposure → deviant peer affiliation). This suggests that parents are more involved in adolescent behavioral development. Therefore, when adolescents are exposed to violence, adolescents with a low level of parental knowledge are more likely to directly exhibit problem behaviors (e.g., violent behaviors). Similarly, Heidari et al. (2014) found that family has an essential factor in the development of high school students' violence, and could directly reduce the incidence of adolescent problem behaviors. Jiang et al. (2016) found that the moderating effect of parental knowledge on the association between risk environment and deviant peer affiliation was only significant for girls.

Interestingly, we found that parental knowledge moderated the indirect relationship between CVE and externalizing problem behavior *via* deviant peer affiliation, and this moderating effect was affected by school climate. These findings supported Hypothesis 2. The positive relationship between CVE and externalizing problem behavior was much stronger for adolescents, who reported lower levels of parental monitoring than for those who reported higher levels of parental monitoring. Specifically, compared with high-level parental knowledge, only low-level parental knowledge had a significant moderating effect on the direct relationship between CVE and externalizing problem behavior. This shows that school factors could decompose the protective effect of parental knowledge to a certain extent (Bernat et al., 2012). Therefore, in future research, especially for students, we should pay attention to the influence of school factors in the mediation mechanism.

## Implications

### Theoretical Implications

First, this research has enriched and perfected the theoretical value of social learning theory and ecosystem theory, to a certain extent, in the interaction of communities, peers, and families in the development of adolescents' behavior. Second, this research proposed a moderated mediation model that reveals the internal mechanism of community violence exposure to externalizing problem behaviors of high school students. This not only further extended our 2017 study, but also further confirmed the mediating effect of deviant peer affiliation. Third, our research further confirmed the moderating effect of parental knowledge, and due to the interference of external factors (e.g., school climate), showed different moderating effects. This has important theoretical value for further research on the impact of community violence exposure on adolescents externalizing problem behaviors in the future.

### Practical Implications

Community violence is a largescale problem with pervasive negative effects on adolescents. Nevertheless, our findings suggest that specific interventions to reduce deviant peer affiliation and increase parental knowledge may reduce externalizing behaviors in some adolescents in this risk environment. After school and evening youth community programs can provide a safe environment and reduce the time adolescents spend in public areas, where they are more vulnerable to the exposure of violence. Furthermore, adolescents in these programs would be interacting with non-deviant peers who are interested in being in the same type of supervised programs.

Our results are consistent with the research of Wang et al. (2016), that is, parental knowledge effectively alleviates the negative influence of the risk environment on adolescents' deviant peer affiliation. Parents could be invited to visit the community programs so that they can get to know the adults and other adolescents with whom their child is spending time, and they could be provided with information about ways to supervise and monitor their children in the context of a violent community environment.

## Limitations

Due to the limitations of our research, the findings of this study should be interpreted with caution. Firstly, causality cannot be determined based on the cross-sectional data. Secondly, all variables were measured by students' self-reports, raising the possibility of biases because of differences in social desirability and shared method variance. However, statistical analysis showed that shared method variance was not a significant problem, and the effect of social desirability may have been attenuated by the use of anonymous questionnaires. Thirdly, the samples taken in this study are low-risk samples, high-risk samples need to be taken in the future to further confirm the research results. Finally, we used a measure of exposure that only included hearing about community violence. In future research, this scale can be further developed to examine other aspects of exposure (i.e., witnessed violence and victimization).

## Conclusion

Compared to our 2017 study, this research has taken a major step forward to better understand the relationship between CVE and externalizing problem behaviors in Chinese adolescents. Our results further indicate that deviant peer affiliation can be used as an important and the closest mediator, through which CVE is related to higher externalizing problem behaviors. Furthermore, parental knowledge can moderate this mediational mechanism: lower parental knowledge has a moderating role in the indirect relationship between heard CVE and externalizing problem behavior *via* deviant peer affiliation after adding school climate as a covariate, thereby suggesting that the negative impacts of CVE can be buffered by parental knowledge to a certain extent. This result further confirms the protective mechanism of parental factors on adolescent development.

## Data Availability Statement

The raw data supporting the conclusions of this article will be made available by the authors, without undue reservation.

## Ethics Statement

The studies involving human participants were reviewed and approved by the Research Ethics Committee of South China Normal University. Written informed consent to participate in this study was provided by the participants' legal guardian/next of kin. Written informed consent was obtained from the individual(s), and minor(s)' legal guardian/next of kin, for the publication of any potentially identifiable images or data included in this article.

## Author Contributions

YZ and YC designed the work. YZ analyzed the data results and drafted the manuscript. WZ revised the manuscript. All authors contributed to the article and approved the submitted version.

## Conflict of Interest

The authors declare that the research was conducted in the absence of any commercial or financial relationships that could be construed as a potential conflict of interest.
